# The combined immunodetection of AP-2α and YY1 transcription factors is associated with *ERBB2 *gene overexpression in primary breast tumors

**DOI:** 10.1186/bcr1851

**Published:** 2008-01-24

**Authors:** Abdelkader Allouche, Gregory Nolens, Annalisa Tancredi, Laurence Delacroix, Julie Mardaga, Viviana Fridman, Rosita Winkler, Jacques Boniver, Philippe Delvenne, Dominique Y Begon

**Affiliations:** 1 Department of Pathology, GIGA-Research, CRCE, University of Liege and CHU of Liege, B23, Avenue de l'Hopital, 3, 4000 Liege, Belgium; 2 Molecular Oncology Laboratory, GIGA-Research, CRCE, University of Liege, B34, Avenue de l'Hopital, 1, 4000 Liege, Belgium; 3 Department of Public Health, Epidemiology and Health Economics, University of Liege, B23, Avenue de l'Hopital, 3, 4000 Liege, Belgium

## Abstract

**Introduction:**

Overexpression of the *ERBB2 *oncogene is observed in about 20% of human breast tumors and is the consequence of increased transcription rates frequently associated with gene amplification. Several studies have shown a link between activator protein 2 (AP-2) transcription factors and *ERBB2 *gene expression in breast cancer cell lines. Moreover, the Yin Yang 1 (YY1) transcription factor has been shown to stimulate AP-2 transcriptional activity on the *ERBB2 *promoter *in vitro*. In this report, we examined the relationships between *ERBB2*, AP-2α, and YY1 both in breast cancer tissue specimens and in a mammary cancer cell line.

**Methods:**

ERBB2, AP-2α, and YY1 protein levels were analyzed by immunohistochemistry in a panel of 55 primary breast tumors. *ERBB2 *gene amplification status was determined by fluorescent *in situ *hybridization. Correlations were evaluated by a χ^2 ^test at a *p *value of less than 0.05. The functional role of AP-2α and YY1 on *ERBB2 *gene expression was analyzed by small interfering RNA (siRNA) transfection in the BT-474 mammary cancer cell line followed by real-time reverse transcription-polymerase chain reaction and Western blotting.

**Results:**

We observed a statistically significant correlation between ERBB2 and AP-2α levels in the tumors (*p *< 0.01). Moreover, associations were found between ERBB2 protein level and the combined high expression of AP-2α and YY1 (*p *< 0.02) as well as between the expression of AP-2α and YY1 (*p *< 0.001). Furthermore, the levels of both AP-2α and YY1 proteins were inversely correlated to *ERBB2 *gene amplification status in the tumors (*p *< 0.01). Transfection of siRNAs targeting AP-2α and AP-2γ mRNAs in the BT-474 breast cancer cell line repressed the expression of the endogenous *ERBB2 *gene at both the mRNA and protein levels. Moreover, the additional transfection of an siRNA directed against the YY1 transcript further reduced the ERBB2 protein level, suggesting that AP-2 and YY1 transcription factors cooperate to stimulate the transcription of the *ERBB2 *gene.

**Conclusion:**

This study highlights the role of both AP-2α and YY1 transcription factors in *ERBB2 *oncogene overexpression in breast tumors. Our results also suggest that high *ERBB2 *expression may result either from gene amplification or from increased transcription factor levels.

## Introduction

The *ERBB2 *oncogene (also known as *HER2*) belongs to the epidermal growth factor receptor gene family and encodes a 185-kDa receptor tyrosine kinase [1]. The *ERBB2 *gene is overexpressed in several human tumors, mostly in breast and ovary carcinomas, where the overexpression is a marker of poor prognosis [2]. Moreover, *ERBB2 *gene overexpression is able to transform cells in culture and to induce mammary tumors in transgenic mice [3]. *ERBB2 *gene-overexpressing tumors are more aggressive due to increased invasive, metastatic, and angiogenic phenotypes [4]. Therefore, elucidating the mechanisms leading to *ERBB2 *gene overexpression is an important step in understanding the pathogenesis of a particularly aggressive subset of breast tumors.

Several laboratories have undertaken the study of the mechanisms leading to the accumulation of high levels of ERBB2 transcript and corresponding protein in breast cancer cells. First, the overexpression of the *ERBB2 *gene has been shown to be partly explained by gene amplification [5]. However, in breast cancer cell lines, regardless of whether the gene is amplified, there is a higher ERBB2 mRNA level per gene copy in overexpressing tumor cells compared with cells with a low ERBB2 expression [6,7]. In addition, we and others have demonstrated that *ERBB2 *overexpression is due to increased transcription rates and not to the stabilization of the mRNA [6,8]. Further experiments, therefore, were needed to identify the activating sequences in the *ERBB2 *promoter, and the molecules that bind them, such as the activator protein 2 (AP-2) transcription factors.

The AP-2 family currently includes five related 50-kDa proteins: AP-2α, AP-2β, AP-2γ [9], AP-2δ [10], and AP-2ε [11]. Several *in vitro *and *in vivo *sets of data have demonstrated a connection between AP-2 transcription factors and *ERBB2 *expression. First, four AP-2 binding sequences were identified in the *ERBB2 *promoter [12-15]. Then, we reported the *in vivo *binding of AP-2 proteins to these sites on the endogenous *ERBB2 *promoter by chromatin immunoprecipitation (ChIP) experiments [13,16]. Moreover, *in vitro *results of transfection experiments have shown that AP-2 factors contribute significantly to the activity of the *ERBB2 *promoter [9,12-16]. In particular, expression of a dominant negative AP-2 protein in mammary cancer cells was shown to result in the inhibition of the transcription from a reporter vector bearing a 6-kb fragment of the *ERBB2 *promoter [13]. Finally, AP-2 transcription factors have been shown to be highly expressed in breast cancer cell lines overexpressing *ERBB2 *[9,14].

AP-2 factors modulate transcription through interactions with several nuclear factors (for example, PARP [17], PC4 [18], CITED2 [19], CITED4 [20], and p300 [21]). Recently, we identified Yin Yang 1 (YY1) as a new cofactor stimulating AP-2 transcriptional activity [16]. YY1 is a multifunctional transcription factor that modulates the expression of a wide variety of genes [22]. It can act as a transcriptional activator or repressor, depending on the context of its binding site within a particular promoter [23] and on other cell type-specific factors [24]. A wide variety of proteins are able to bind to YY1, indicating that protein-protein interactions are important for its activity. Among these proteins, YY1 interacts with AP-2α through a domain highly conserved in AP-2γ [25]. Moreover, YY1 enhances AP-2α, AP-2β, and AP-2γ transcriptional activity on the *ERBB2 *promoter in breast cancer cells [16]. ChIP experiments also showed that the YY1 protein is recruited on the endogenous *ERBB2 *promoter only when a member of the AP-2 protein family is present [16].

The aim of this study was to characterize better the relationship between the overexpression of *ERBB2 *oncogene and AP-2α transcription factor in primary breast tumors and to determine whether the expression level of the YY1 cofactor could play a role in the association between the expression of AP-2α and *ERBB2*. In this study, we first demonstrated that the expression of these proteins is positively correlated in breast cancer tissues. These results were further associated with *ERBB2 *gene amplification status and then corroborated by a functional analysis using small interfering RNA (siRNA) transfected in a mammary cancer cell line. Altogether, our data indicate that *ERBB2 *gene amplification or increased levels of transcription factors may lead to a pathologically high level of ERBB2 transcript and protein in breast cancer.

## Materials and methods

### Tissue samples

A series of 55 primary tumors from breast cancer patients diagnosed between 2002 and 2004 at the University Hospital of Liege, Belgium, was analyzed. The mean age of the patients was 61.9 years and the median was 59.0 years (range: 38.0 to 88.0 years). The clinicopathological data of the patients are summarized in Table 1. The tumor samples were fixed in 10% buffered formalin and embedded in paraffin. The histological diagnosis was confirmed by reviewing the original sections of the primary tumors. All of the tumors were simultaneously evaluated for histological type and grade by senior pathologists. The most representative blocks were selected and cut into new 5-μm-thick sections for immunohistochemical analyses. The study was approved by the local ethics committee at the Liege University Hospital.

**Table 1:** Clinicopathological data of the patients and their relationships with ERBB2 expression

Characteristic	*n*	(Percentage)	ERBB2 expression (percentage)				*P *value
			(0/1+/2+)		(3+)		
Number of patients	55	(100)	40	(73)	15	(27)	
Tumor size							NS
T_1_	20	(36)	16	(80)	4	(20)	
T_2_	29	(53)	20	(69)	9	(31)	
T_3_	6	(11)	4	(67)	2	(33)	
							
Lymph node status							NS
Negative	33	(60)	24	(73)	9	(27)	
Positive	22	(40)	16	(73)	6	(27)	
							
Grade							
Not determined	3	(5)	/				
I	12	(22)	29	(83)	6	(17)	0.060
II	23	(42)					
III	17	(31)	10	(59)	7	(41)	
							
Histological type							NS
Ductal	40	(73)	28	(70)	12	(30)	
Lobular	7	(13)	5	(71)	2	(29)	
Other	8	(14)	7	(87)	1	(13)	
							
ER status							0.035
Positive	42	(76)	34	(81)	8	(19)	
Negative	13	(24)	6	(46)	7	(54)	
							
PR status							0.022
Positive	32	(58)	27	(84)	5	(16)	
Negative	23	(42)	13	(57)	10	(43)	
							
Menopausal status							NS
Premenopausal	9	(16)	8	(89)	1	(11)	
Postmenopausal	46	(84)	32	(70)	14	(30)	
							
Ki67							0.018
Low	29	(53)	25	(86)	4	(14)	
High	26	(47)	15	(58)	11	(42)	
							
p53							0.052
Low	46	(84)	36	(78)	10	(22)	
High	9	(16)	4	(44)	5	(56)	

ER, estrogen receptor; grade, Elston-Ellis modification of Bloom grade; NS, not significant; PR, progesterone receptor.

### Immunohistochemistry

Sections of breast biopsy specimens underwent immunoperoxidase staining using antibodies directed against AP-2α (1:100) (#39001; Active Motif, Carlsbad, CA, USA), against YY1 (1:50) (H-10; Santa Cruz Biotechnology, Inc., Santa Cruz, CA, USA), or against ERBB2 (1:250) (A0485; Dako A/S, Glostrup, Denmark). The sections were deparaffinized in xylene and rehydrated in methanol. Endogenous peroxidases were blocked by 5% H_2_O_2 _treatment. For better antigen retrieval, the samples were boiled either in a microwave oven for 3 × 5 minutes in citrate buffer (AP-2α and YY1) or in a water bath at 99°C in EDTA (ethylenediaminetetraacetic acid) buffer for 40 minutes (ERBB2). Samples were then washed with phosphate-buffered saline-Tween (pH 7.2; 1.5%) and incubated with the primary antibody at room temperature for 30 minutes (AP-2α and YY1) or 1 hour (ERBB2). After washings, the revelation was performed with the use of appropriate secondary antibodies and the LSAB2 system (AP-2α and YY1; Dako A/S) or the EnVision kit (ERBB2; Dako A/S) according to the supplier's recommendations. Immunoreactivity was visualized by a treatment with diaminobenzidine (Sigma-Aldrich, St. Louis, MO, USA), and the slides were counterstained with Mayer's hematoxylin.

For statistical analyses of AP-2α and YY1 immunoreactivity, the percentage distribution of stained tumor cell nuclei in the sample was divided into low (<80%) or high (≥ 80%) expression groups according to Pellikainen and colleagues [26]. ERBB2 scoring was performed according to the recently proposed guidelines from the American Society of Clinical Oncology (ASCO) and the College of American Pathologists (CAP) [27]. Pathological ERBB2 overexpression (3+) was detected in 27% of tumors. There was a significant statistical association between ERBB2 overexpression and Ki67 immunostaining, and an inverse relationship was demonstrated with estrogen receptor and progesterone receptor status (Table 1). Furthermore, a trend toward a direct link between *ERBB2 *overexpression and both p53 expression and histological grade III was observed.

### Fluorescent *in situ *hybridization

Fluorescent *in situ *hybridization (FISH) was performed with the INFORM HER-2/neu probe (approved by the U.S. Food and Drug Administration) and the BenchMark XT automated system (Ventana Medical Systems, Inc., Tucson, AZ, USA) according to the supplier's recommendations. A minimum of 50 cell nuclei were counted, and gene amplification was considered as present when an average of more than six *ERBB2 *gene copies per cell was observed [27].

### Statistics

The statistical analyses were carried out with a χ^2 ^test for categorical variables at a *p *value of less than 0.05 for significance by using Statistica software (StatSoft, Inc., Tulsa, OK, USA).

### Cell line

The BT-474 human mammary carcinoma cells were purchased from the American Type Culture Collection (Manassas, VA, USA) and cultured in the RPMI 1640 medium supplemented with 10% (vol/vol) fetal bovine serum, 2 mM glutamine, and 100 μg/mL penicillin/streptomycin (all from Cambrex Bio Science Verviers S.p.r.l., Verviers, Belgium).

### Small interfering RNAs

Cells were transfected (a) on days 0 and 2 by 150 nM siRNA directed against AP-2α and/or AP-2γ transcripts as indicated or (b) on day 0 by 30 nM siRNA against YY1, or 100 nM combined siRNAs against AP-2α and AP-2γ transcripts, or both as indicated. As control, cells were transfected by either an siRNA against luciferase mRNA [28] or a negative control siRNA OR-0030-neg05 from Eurogentec S.A. (Seraing, Belgium). Total RNA was extracted after 2 to 4 days of treatment. Real-time reverse transcription-polymerase chain reaction (RT-PCR) for AP-2α, AP-2γ, ERBB2, and β2-microglobulin (standard gene) transcripts were performed on 1 μg of total extracted RNA. The standardized transcript levels were reported to the values obtained in cells transfected with the control siRNA. The RT-PCR analysis was performed on an ABI Prism 7000 apparatus (Applied Biosystems, Foster City, CA, USA) using standard protocol. Western blot analysis was performed on proteins extracted after 1 or 3 days of treatment as indicated. The antibodies used for Western blot were 3B5 for AP-2α, 6E4/4 for AP-2γ, H-10 for YY1, and C-19 for Ku70 (all purchased from Santa Cruz Biotechnology, Inc.) and a rabbit antibody for ERBB2 (06–562; Upstate, now part of Millipore Corporation, Billerica, MA, USA). The sequences of the siRNAs and the RT-PCR primers (all purchased from Eurogentec S.A.) are presented in Table 2.

**Table 2:** Sequences of small interfering RNAs and primers for reverse transcription-polymerase chain reaction

	Sequence 5'-3'	Location
siRNAs		
siAP-2α ss	CCGAAUUUCCUGCCAAAGCdTdT	
siAP-2α as	GCUUUGGCAGGAAAUUCGGdTdT	
siAP-2γ ss	UUAAAUAUUCUGCCACUGGdTdT	
siAP-2γ as	CCAGUGGCAGAAUAUUUAAdTdT	
siYY1 ss	GAACUCACCUCCUGAUUAUdTdT	
siYY1 as	AUAAUCAGGAGGUGAGUUCdTdT	
RT-PCR primers		
AP-2α forward	AGCTGAATTTCTCAACCGACAAC	1,013 (exon 5)
AP-2α reverse	TAGCCAGGAGCATGTTTTTTCTT	1,083 (exon 6)
AP-2γ forward	CAGAAGAGCCAAATCGAAAAATG	1,041 (exons 5–6)
AP-2γ reverse	ATTCAACCCAATCTTGTCCAACTT	1,107 (exon 6)
*ERBB2 *forward	CTGAACTGGTGTATGCAGATTGC	2,617 (exon 20)
*ERBB2 *reverse	TTCCGAGCGGCCAAGTC	2,699 (exon 21)

as, antisense strand; RT-PCR, reverse transcription-polymerase chain reaction; siRNA, small interfering RNA; ss, sense strand.

## Results

### Combined high AP-2a and YY1 levels are associated with expression of ERBB2 in breast cancer tissue samples

**Figure 1:**
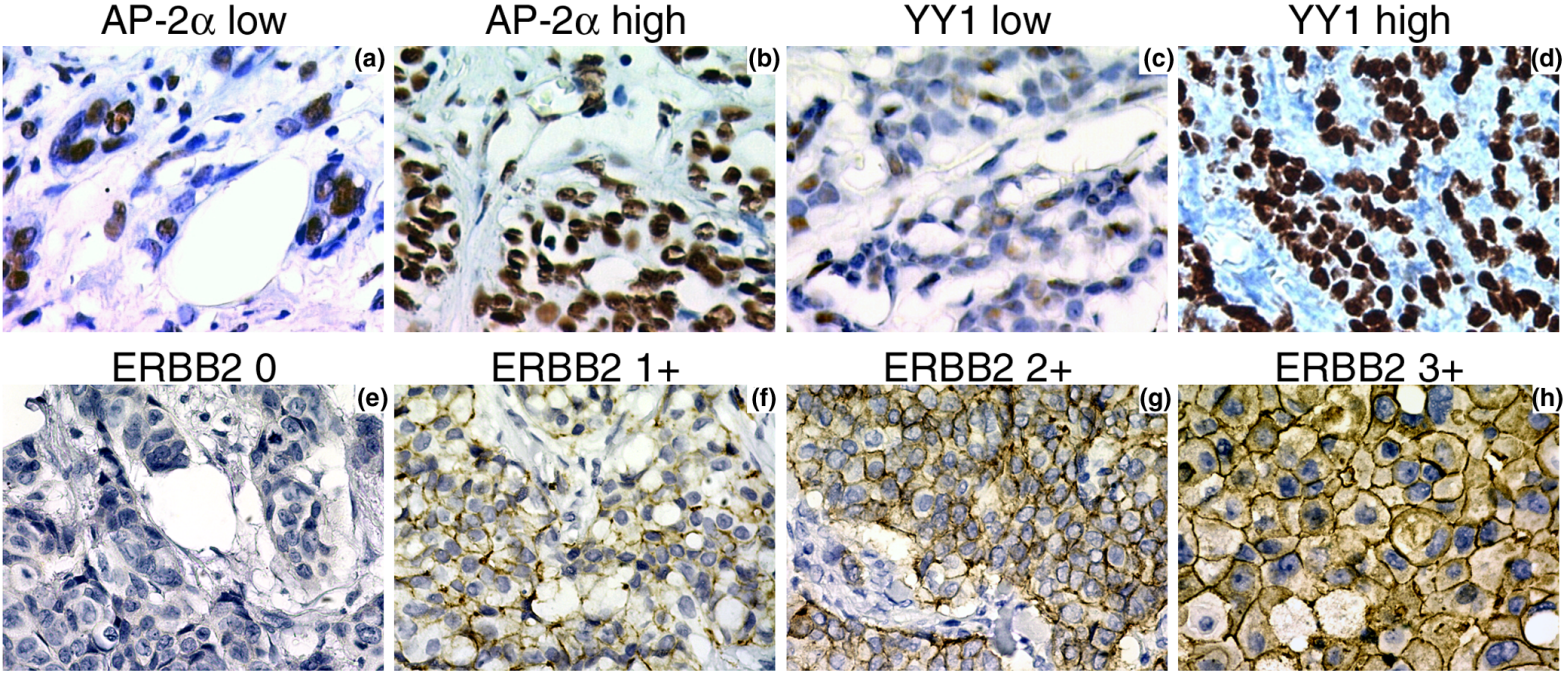
Detection of activator protein 2 alpha (AP-2α), Yin Yang 1 (YY1), and ERBB2 by immunohistochemistry in breast tumors. **(a) **Case with low immunoreactivity for AP-2α. **(b) **Tumor sample expressing high level of AP-2α protein in more than 80% of the nuclei.**(c) **Tumor with low immunoreactivity for YY1. **(d) **Case expressing high level of YY1 protein in more than 80% of the nuclei. **(e) **Case with no ERBB2 membrane staining, scored as IHC 0. **(f) **Tumor with partial weak membrane staining, scored as IHC 1+. **(g) **Case with ERBB2 score of 2+. **(h) **Tumor with thick circumferential ERBB2 membrane staining, scored as IHC 3+.

We first detected levels of ERBB2, AP-2α, and YY1 proteins by immunohistochemistry (IHC) in tumor specimens from 55 cases of breast carcinomas (Tables 1 and 3). Representative examples of the staining patterns obtained for ERBB2 receptor and the transcription factors AP-2α and YY1 are shown in Figure 1. The AP-2α and YY1 proteins were detected mainly in the nuclear compartment, while cytoplasmic staining was rare and weak (Figure 1a–d). We scored the AP-2α and YY1 levels as low or high regarding the percentage of stained nuclei according to Pellikainen and colleagues [26] (Figure 1). High AP-2α and YY1 protein levels were seen in 42% and 45% of breast carcinomas, respectively. For ERBB2, we considered only membranous staining (Figure 1e–h). Scoring was carried out according to the ASCO/CAP guidelines [27].

**Table 3:** Associations between ERBB2 and AP-2α and/or YY1 levels determined by immunohistochemistry

		ERBB2 expression (percentage)						
	*n*	0/1+		2+		3+		*P *value
AP-2α expression								0.003
Low	32	19	(59)	5	(16)	8	(25)	
High	23	4	(17)	12	(52)	7	(31)	
								
YY1 expression								0.318
Low	30	15	(50)	7	(23)	8	(27)	
High	25	8	(32)	10	(40)	7	(28)	
								
Combination								0.016
AP-2α lo/YY1 lo	26	14	(54)	4	(15)	8	(31)	
AP-2α lo/YY1 hi or AP-2α hi/YY1 lo	10	6	(60)	4	(40)	0	(0)	
AP-2α hi/YY1 hi	19	3	(16)	9	(47)	7	(37)	

AP-2α, activator protein 2 alpha; hi, high expression; lo, low expression; YY1, Yin Yang 1.

We then analyzed the correlations between the levels of ERBB2, AP-2α, and YY1 proteins in the tumors. Our statistical analyses showed that ERBB2 expression was significantly associated with a high AP-2α transcription factor level (*p *= 0.003) (Table 3). Accordingly, 83% of the tumors with high AP-2α level had a 2+ or 3+ IHC score for ERBB2 protein and only 17% had a low ERBB2 expression (0 to 1+). On the other hand, 83% of the tumors with ERBB2 low expression had a low level of AP-2α protein. In contrast, no association between ERBB2 and YY1 expression was observed (Table 3). However, there was a significant association of ERBB2 protein level with combined overexpression of AP-2α and YY1 transcription factors (*p *≤ 0.02) (Table 3). Indeed, among the 19 cases presenting high levels of both AP-2α and YY1, 84% had an ERBB2 2+ or 3+ score (Table 3).

### Levels of both AP-2a and YY1 proteins are inversely associated with ERBB2 amplification status in primary breast cancer samples

FISH demonstrated *ERBB2 *gene amplification in 11 cases of breast cancer. All of these had an ERBB2 3+ score in IHC. Moreover, out of the 15 ERBB2 3+ cases, 11 were FISH-positive, 2 showed no *ERBB2 *amplification, 1 was borderline with an average of 4 copies of *ERBB2 *gene per cell, and 1 was undetermined due to lack of material for FISH testing. Interestingly, the 2 cases of ERBB2 3+ immunostaining without gene amplification and the borderline case showed high AP-2α and YY1 levels. On the other hand, all the ERBB2 3+ cases with low levels of both AP-2α and YY1 showed *ERBB2 *gene amplification. Furthermore, when considering the entire ERBB2 expressing group (1+, 2+, 3+), we observed a significant inverse correlation between *ERBB2 *FISH status on one hand and AP-2α and YY1 levels on the other hand (*p *= 0.017 and 0.029, respectively) (Table 4). In particular, 80% of the cases with high levels of both AP-2α and YY1 proteins did not present *ERBB2 *gene amplification (*p *= 0.006) (Table 4). These results suggest that when the *ERBB2 *gene is not amplified, the *ERBB2 *expression may rely partially on AP-2α and YY1 transcription factor levels. Accordingly, considering only the FISH-negative cases (n = 34), the correlation between ERBB2 and AP-2α levels was higher than in the whole group (*r *= 0.67, *p *< 0.001 compared with *r *= 0.31, *p *= 0.022 in the whole group). Interestingly, the percentage of ERBB2 2+3+/AP-2α-low cases decreased (12% in the FISH-negative group compared with 24% in the entire group) (Table 5). Similarly, the percentage of ERBB2-positive/AP-2α-low/YY1-low cases diminished (from 22% in the whole group to 9% in the FISH-negative group) (Table 5). These results indicate that high *ERBB2 *expression may result either from gene amplification or from increased transcription factor levels.

**Table 4:** Inverse associations between FISH results and AP-2α and/or YY1 levels in ERBB2 expressing group (1+, 2+, 3+)

		*ERBB2 *FISH (percentage)				
	*n*	Negative		Positive		*P *value
AP-2α expression						0.017
Low	12	4	(33)	8	(67)	
High	18	15	(83)	3	(17)	
						
YY1 expression						0.029
Low	14	6	(43)	8	(57)	
High	16	13	(81)	3	(19)	
						
Combination						0.006
AP-2α lo/YY1 lo	11	3	(27)	8	(73)	
AP-2α lo/YY1 hi or AP-2α hi/YY1 lo	4	4	(100)	0	(0)	
AP-2α hi/YY1 hi	15	12	(80)	3	(20)	

AP-2α, activator protein 2 alpha; FISH, fluorescent *in situ *hybridization; hi, high expression; lo, low expression; YY1, Yin Yang 1.

**Table 5:** Associations between ERBB2 and AP-2α and/or YY1 levels (immunohistochemistry) in the FISH-negative group

		ERBB2 expression (percentage)						
	*n*	0/1+		2+		3+		*P *value
AP-2α expression								<0.001
Low	18	14	(78)	4	(22)	0	(0)	
High	16	2	(12)	11	(69)	3	(19)	
								
YY1 expression								0.106
Low	16	10	(63)	6	(37)	0	(0)	
High	18	6	(33)	9	(50)	3	(17)	
								
Combination								0.015
AP-2α lo/YY1 lo	13	10	(77)	3	(23)	0	(0)	
AP-2α lo/YY1 hi or AP-2α hi/YY1 lo	8	4	(50)	4	(50)	0	(0)	
AP-2α hi/YY1 hi	13	2	(15)	8	(62)	3	(23)	

AP-2α, activator protein 2 alpha; FISH, fluorescent *in situ *hybridization; hi, high expression; lo, low expression; YY1, Yin Yang 1.

### Inhibition of AP-2 and YY1 downregulates ERBB2 expression *in vitro*

**Figure 2:**
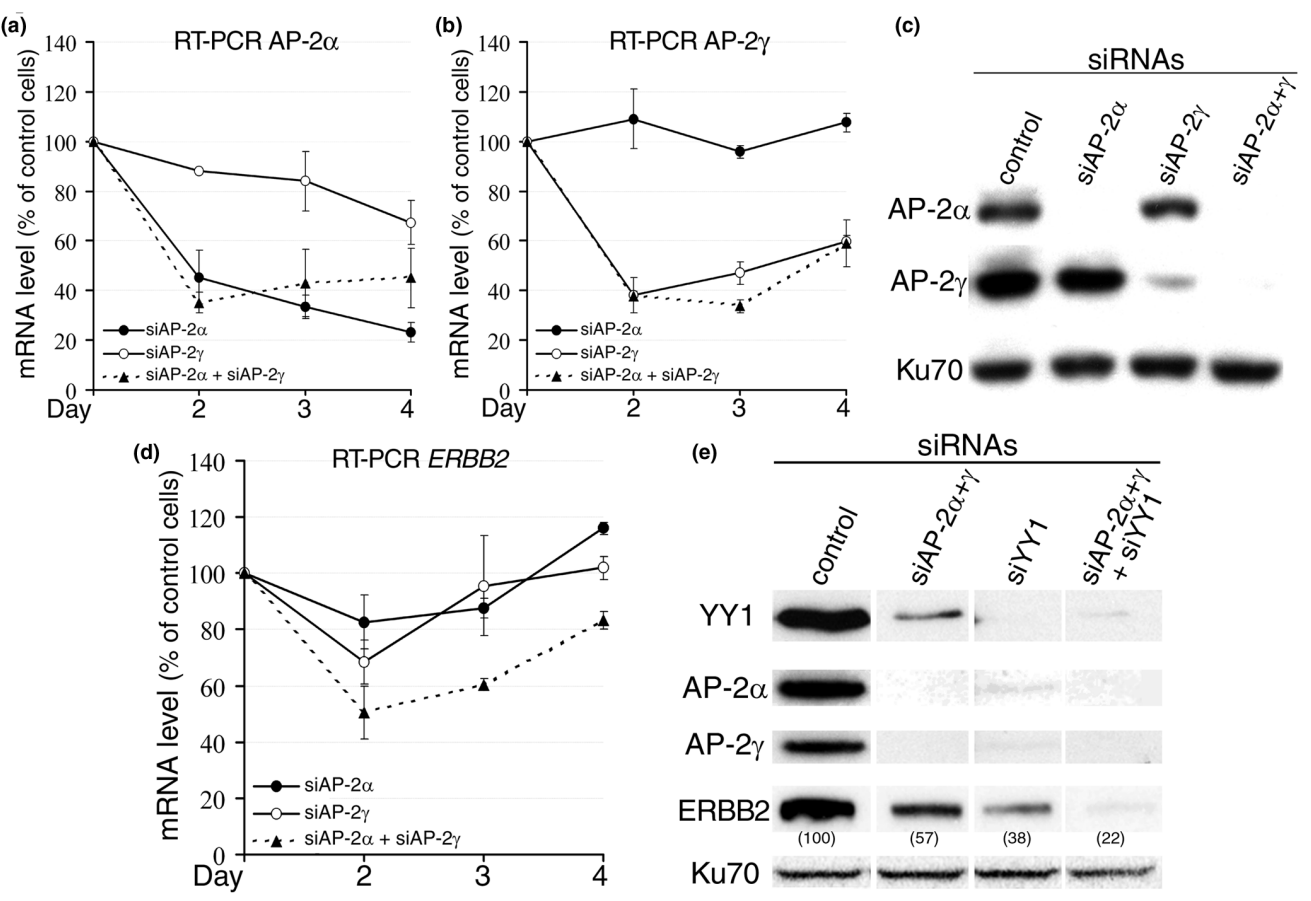
Suppression of AP-2α, AP-2γ, and YY1 expression downregulates ERBB2 transcript levels in BT-474 cells. **(a) **Cells were transfected on days 0 and 2 by small interfering RNAs (siRNAs) directed against AP-2α (*siAP-2α*) and/or AP-2γ (*siAP-2γ*) or against luciferase mRNA as control. Graphic shows real-time reverse transcription-polymerase chain reaction (RT-PCR) for AP-2α transcripts on total RNA extracted after 2, 3, or 4 days of treatment with indicated siRNAs. Results are presented as percentages of mRNA level as compared with control cells transfected with luciferase siRNA. Data are means ± standard deviation of three experiments. **(b) **RT-PCR for AP-2γ transcripts on total RNA. Cells were transfected like in (a). **(c) **Detection by Western blotting of AP-2α and AP-2γ levels at day 3. Ku70 protein served as control. Cells were transfected like in (a).**(d) **RT-PCR for ERBB2 transcripts on total RNA. Cells were transfected like in (a). **(e) **Cells were transfected on day 0 with 30 nM siRNA directed against YY1 (*siYY1*) or 100 nM combined siRNAs against AP-2α and AP-2γ transcripts (siAP-2α+γ) or both as indicated. Control cells were transfected with a commercially available negative control siRNA (*control*). Proteins extracted after 24 hours of treatment were detected by Western blotting. Ku70 protein served as control for the protein amount charged on the gel. The percentage of ERBB2 protein level compared with transfection of control siRNA is shown in brackets. AP-2, activator protein 2; YY1, Yin Yang 1.

After these correlation results in breast cancer specimens, we sought to study these relationships on the functional side. Although the AP-2 family is known to activate the *ERBB2 *promoter in reporter vectors, the effect of these transcription factors on the expression of the endogenous *ERBB2 *gene has not been clearly established. To find out whether AP-2 factors do contribute functionally to *ERBB2 *overexpression *in vivo*, we measured ERBB2 mRNA levels in breast cancer cells in which the expressions of AP-2α and AP-2γ were downregulated by siRNAs. BT-474 breast cancer cells overexpressing *ERBB2*, both through amplification and enhanced transcription, were transfected with AP-2α and AP-2γ siRNAs, both independently and in combination for several days. Two different siRNAs against AP-2α and three distinct siRNAs against AP-2γ were first tested (data not shown). The best one for each target, as tested by Western blotting, was further used in the study. First, AP-2α and AP-2γ transcript levels were quantified by real-time RT-PCR in the cells transfected by the AP-2 siRNAs and reported to transfection of a control siRNA. Transfection of AP-2α siRNA alone inhibited AP-2α expression (Figure 2a) but did not modify the AP-2γ transcript level (Figure 2b). Comparable results were obtained in cells transfected with AP-2γ siRNA alone (Figure 2a,b). Moreover, transfection of both AP-2α and AP-2γ siRNAs induced a decrease in both mRNA levels (Figure 2a,b). Similar results were obtained at the protein level (Figure 2c, Western blotting). Markedly, 3 days after treatment with the combination of AP-2α and AP-2γ siRNAs, both factors were undetectable (Figure 2c, lane 4), demonstrating that the siRNAs specifically inhibited the expression of their targets. We then quantified ERBB2 transcript level by real-time RT-PCR in those cells transfected by the siRNAs. Either the AP-2α siRNA or the AP-2γ siRNA alone produced a small transient downregulation of the ERBB2 transcript level (Figure 2d). Interestingly, transfection of both AP-2α and AP-2γ siRNAs induced a significant decrease in the endogenous ERBB2 mRNA level (Figure 2d). This result was also obtained with another set of siRNAs against AP-2α and AP-2γ (data not shown). Moreover, the inhibition was seen at the protein level (Figure 2e, lane 2). Indeed, we observed a reduction of ERBB2 protein level to 57% of control upon transfection of both siRNAs directed against AP-2α and AP-2γ. Going deeper, we further added an siRNA directed against YY1 and observed that the ERBB2 protein level could be decreased even further to 22% of control by this combination of siRNAs (Figure 2e, lane 4). These results are strong evidence that AP-2 and YY1 transcription factors effectively participate in *ERBB2 *expression in breast cancer cells.

### AP-2 and YY1 protein levels are correlated both in mammary cancer cell line and tissue specimens

Surprisingly, we also observed a strong association between AP-2α and YY1 expression levels in the primary breast tumors (*p *< 0.001) (Table 6). Indeed, the majority of the tumors contained either low levels (26 cases) or high amounts (19 cases) of both proteins. In contrast, there were only 10 cases (18%) with a high level of either AP-2α or YY1 alone (Table 6). Next, we analyzed the relationship between these factors in a breast cancer cell line. We observed that transfection of an siRNA directed against YY1 reduced both AP-2α and AP-2γ protein levels (Figure 2e, lane 3). Additionally, the combination of siRNAs directed against both AP-2α and AP-2γ also diminished the YY1 protein level (Figure 2e, lane 2). These results suggest that, besides the cooperation between AP-2 and YY1 transcription factors on the *ERBB2 *promoter, there is an intricate relationship between the expressions of these proteins.

**Table 6:** Association between AP-2α and YY1 levels determined by immunohistochemistry on breast tumor specimens

		AP-2α expression (percentage)				
			
	*n*	Low		High		*P *value
YY1 expression						<0.001
Low	30	26	(87)	4	(13)	
High	25	6	(24)	19	(76)	

AP-2α, activator protein 2 alpha; YY1, Yin Yang 1.

## Discussion

Multiple *in vitro *and *in vivo *data have demonstrated a link between AP-2 transcription factors and the *ERBB2 *oncogene. The goals of this study were to characterize better the relationship between the overexpressions of *ERBB2 *and AP-2α in primary breast tumors and to analyze the implication of the YY1 protein as a cofactor of AP-2. Although the number of tumors analyzed in this study was quite small, it should be stressed that our observations in tissue samples were further corroborated by data from the functional *in vitro *experiments.

Previous immunohistochemical studies on AP-2 and ERBB2 expression in primary breast tumors have reported controversial conclusions [26,29-31]. Indeed, whereas three studies showed a direct correlation between the levels of one or two AP-2 transcription factor family members and *ERBB2 *gene expression [26,29,31], a fourth one revealed no correlation [30]. Moreover, Turner and colleagues [31] showed that the combination of AP-2α and AP-2γ expressions correlated to ERBB2 expression. It should be noted that these multiple studies used different antibodies which sometimes did not discriminate between the diverse members of the AP-2 family. In the present study, we focused on the AP-2α member and showed a strongly significant correlation between AP-2α and ERBB2 protein levels. Moreover, we demonstrated that *ERBB2 *endogenous expression is inhibited by the downregulation of both AP-2α and AP-2γ in a breast cancer cell line. These results and the fact that AP-2 factors bind the endogenous *ERBB2 *gene promoter [13,16] strongly suggest that AP-2 factors do effectively contribute to *ERBB2 *overexpression in breast cancer cells. Our siRNA results further indicate that both AP-2α and AP-2γ are required for *ERBB2 *overexpression, as already suggested in a study on breast cancer tissues by Turner and colleagues [31].

In the present study, *ERBB2 *expression was also associated with combined high levels of AP-2α and its partner YY1 both in primary breast tumors, markedly in the absence of *ERBB2 *gene amplification, and in a breast cancer cell line. The fact that YY1 needs AP-2 to be recruited on the endogenous *ERBB2 *proximal promoter [16] could explain why no direct relationship between YY1 and ERBB2 levels was seen in the tumors and highlights the importance of the combination of high levels of AP-2α and YY1 for *ERBB2 *expression. To the best of our knowledge, our study is the first to report both data in primary tumors and functional results on breast cancer cells regarding the role of AP-2α transcription factor and one of its cofactors on *ERBB2 *gene expression. Further investigations, however, are needed to determine whether breast tumors with ERBB2 2+ or 3+ IHC score without gene amplification but with high levels of AP-2α and YY1 have a particular behaviour regarding clinical outcome and response to Herceptin treatment.

Recently, Li and colleagues [32] proposed that AP-2α overexpression in breast cancer cells is the consequence of a stabilization of the protein resulting from a defective proteasomal degradation, leading to an increased *ERBB2 *gene expression. Our results further suggest that *ERBB2 *overexpression in breast tumors results not only from a high expression of AP-2α but also from the concomitant high expression of YY1. Indeed, we have previously shown that YY1 is able to enhance AP-2α, AP-2β, and AP-2γ transcriptional activity *in vitro *and that it is recruited via AP-2 to the *ERBB2 *endogenous promoter in a breast cancer cell line [16]. Moreover, in the present study, we observed a strong correlation between AP-2α and YY1 protein levels in the primary breast tumors and a decrease in the AP-2α and AP-2γ levels following transfection of an siRNA targeting YY1. These data suggest that there may be a two-sided contribution of YY1 for *ERBB2 *expression. On the one hand, YY1 increases the AP-2α and AP-2γ protein levels, and on the other hand, YY1 stimulates AP-2α and AP-2γ transcriptional activity. This view fits well with the additive effect of the siRNAs targeting AP-2α, AP-2γ, and YY1 for inhibiting ERBB2 endogenous level.

Another finding of this study was the positive correlation between AP-2α and YY1. Interestingly, AP-2α promoter is dependent upon an initiator element for proper transcription, and we noticed a potential YY1 binding site at the transcription start site of the AP-2α gene (position +4) [33]. Since YY1 can act as an initiator factor [24], this could explain the effect of YY1 siRNA on the AP-2α level. We also found a perfect AP-2 consensus binding site on the YY1 promoter at -236 base pairs upstream from the transcription start site [34]. Further studies are needed to determine whether these binding sites are functional and may explain the relationship between AP-2α and YY1 levels.

## Conclusion

We demonstrated, for the first time, the implication of AP-2α in combination with its cofactor, YY1, in *ERBB2 *oncogene overexpression in breast tumors both *in vitro *and in breast cancer tissue specimens. Moreover, we showed that at least two mechanisms can lead to pathological *ERBB2 *overexpression in breast cancer: *ERBB2 *gene amplification and increased transcription through high levels of transcriptional activators, such as AP-2α and YY1. We hope that these data open a new way in the research field of *ERBB2 *overexpression and its pathogenic role in breast tumors.

## Abbreviations

AP-2 = activator protein 2; ASCO = American Society of Clinical Oncology; CAP = College of American Pathologists; ChIP = chromatin immunoprecipitation; FISH = fluorescent *in situ *hybridization; IHC = immunohistochemistry; RT-PCR = reverse transcription-polymerase chain reaction; siRNA = small interfering RNA; YY1 = Yin Yang 1.

## Competing interests

The authors declare that they have no competing interests.

## Authors' contributions

AA selected the histological specimens and performed and scored immunohistochemistry. GN performed siRNA transfection experiments and Western blotting. AA and GN contributed equally to this work. AT carried out the statistical analyses. LD performed siRNA transfection experiments, Western blotting, and real-time RT-PCR. JM and JB made substantial contributions to manuscript revisions. VF scored the ERBB2 immunohistochemistry. RW participated in the study design, revised the manuscript, and provided important intellectual support. PD conceived of the study, scored FISH on the histological specimens, revised the manuscript, and provided important intellectual support. DYB participated in the study design, coordination, and interpretation of the results and drafted and finalized the manuscript. All authors read and approved the final manuscript.
